# Supplementary material for: Venomix: a simple bioinformatic pipeline for identifying and characterizing toxin gene candidates from transcriptomic data
Source: PeerJ. 2018 Jul 31;6:e5361. doi: 10.7717/peerj.5361 (PMC6074769; doi:10.7717/peerj.5361)
Supplement: Supplemental Information 3 [file peerj-06-5361-s003.gz › FinalOutput_E-20/Zinc_metalloproteinase-disintegrin-like_ammodytagin_1/finaltree.pdf]

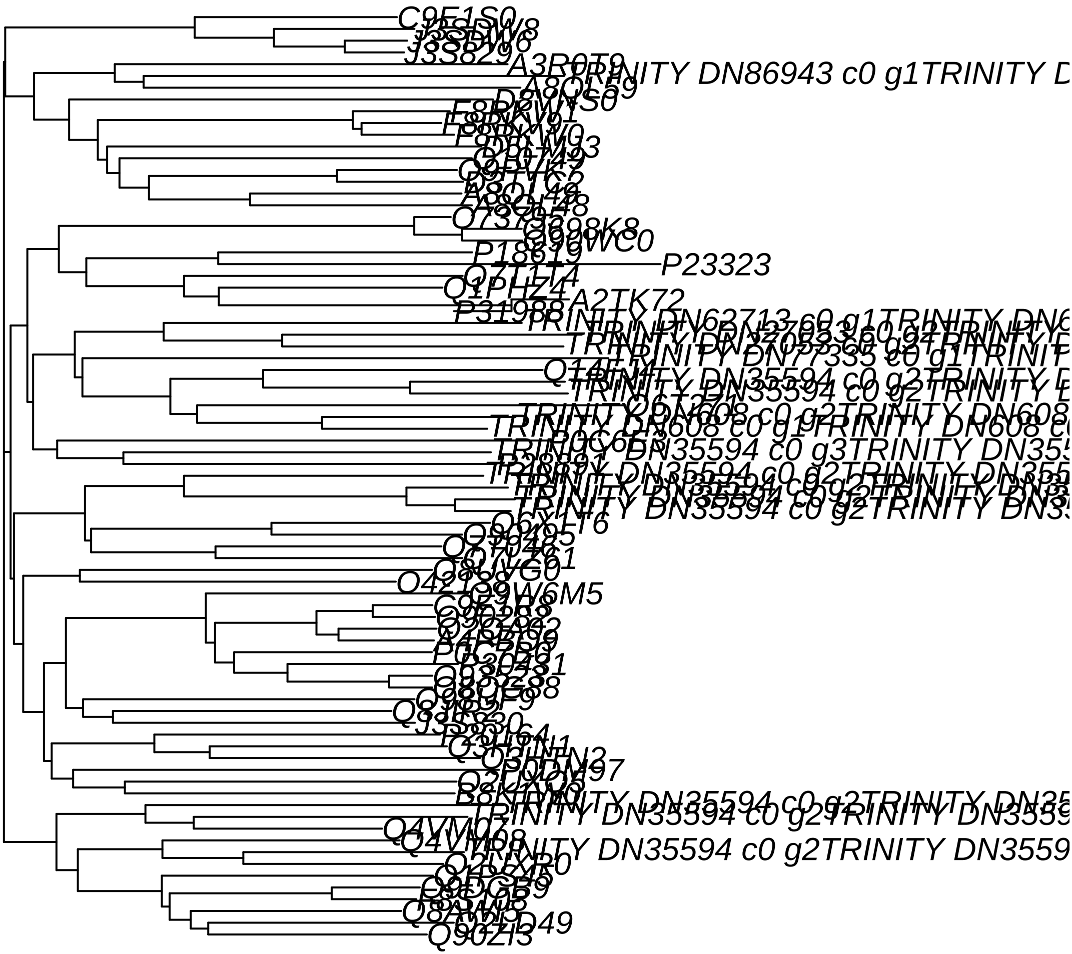
C9E1S0
J3S829
A3R0T9
TRINITY DN86943 c0 g1TRINITY D
P23323
P18619
Q1PHZ4
A2TK72
O42129
Q4VM08
TRINITY DN35594 c0 g2TRINITY DN3559
Q90ZI3
